# The Uncertain Geographic Context Problem in the Analysis of the Relationships between Obesity and the Built Environment in Guangzhou

**DOI:** 10.3390/ijerph15020308

**Published:** 2018-02-10

**Authors:** Pengxiang Zhao, Mei-Po Kwan, Suhong Zhou

**Affiliations:** 1 Department of Land Surveying and Geo-Informatics, The Hong Kong Polytechnic University, Hong Kong, China; peng.x.zhao@polyu.edu.hk; 2 Department of Geography and Geographic Information Science, University of Illinois at Urbana-Champaign, Natural History Building, MC-150, 1301 W Green Street, Urbana, IL 61801, USA; mpk654@gmail.com; 3 Department of Human Geography and Spatial Planning, Faculty of Geosciences, Utrecht University, P.O. Box 80125, 3508 TC Utrecht, The Netherlands; 4 School of Geography and Planning, Sun Yat-sen University, Guangzhou 510275, China; 5 Guangdong Key Laboratory for Urbanization and Geo-simulation, Guangzhou 510275, China

**Keywords:** obesity, built environment, activity space, regression analysis, UGCoP

## Abstract

Traditionally, static units of analysis such as administrative units are used when studying obesity. However, using these fixed contextual units ignores environmental influences experienced by individuals in areas beyond their residential neighborhood and may render the results unreliable. This problem has been articulated as the uncertain geographic context problem (UGCoP). This study investigates the UGCoP through exploring the relationships between the built environment and obesity based on individuals’ activity space. First, a survey was conducted to collect individuals’ daily activity and weight information in Guangzhou in January 2016. Then, the data were used to calculate and compare the values of several built environment variables based on seven activity space delineations, including home buffers, workplace buffers (WPB), fitness place buffers (FPB), the standard deviational ellipse at two standard deviations (SDE2), the weighted standard deviational ellipse at two standard deviations (WSDE2), the minimum convex polygon (MCP), and road network buffers (RNB). Lastly, we conducted comparative analysis and regression analysis based on different activity space measures. The results indicate that significant differences exist between variables obtained with different activity space delineations. Further, regression analyses show that the activity space delineations used in the analysis have a significant influence on the results concerning the relationships between the built environment and obesity. The study sheds light on the UGCoP in analyzing the relationships between obesity and the built environment.

## 1. Introduction

Much health and geographic research has examined how the physical and social environment affects people’s health. As a major risk factor for a series of health problems, including heart disease, stroke, cardiovascular disease, diabetes, sleep apnea, osteoarthritis, and some cancers, obesity has become a major public health concern worldwide [1,2]. Excessive energy intake and a lack of physical activity have been identified as major risk factor for obesity at the individual level [3]. In this context, disparities in obesity prevalence can be attributed to people’s food environment and built environment. In past studies, the food environment is often regarded as part of the built environment. Recent extensive studies indicate that the built environment has a potential influence on obesity [4,5,6,7]. For instance, Li et al., examined the relationships between weight, and physical and social environments, using multi-level regression analysis based on social survey data [8]. Townshend and Lake investigated how the built environment influences physical activity and dietary behaviors [9].

The built environment is broadly defined as human-made facilities and infrastructure used to support human activity, including roads, public transportation, buildings, restaurants, supermarkets, and other amenities, which influence obesity-related behaviors [10,11]. Many studies have examined the relationships between the built environment and obesity [11,12,13,14]. However, a fundamental methodological issue in research on this relationship at the individual level remains: different delineations of geographic context may lead to different values of the contextual variables, and may thus influence the results concerning the relationships between the contextual variables and the health behavior or outcome being studied. 

In most of the previous studies on obesity, the influence of the built environment on obesity was examined using geographic context based on people’s residential neighborhoods or administrative units, such as census tracks or postcode areas. For instance, Frank et al. used a 1 km network distance buffer around each participant’s place of residence to develop objective measures of land use mix, residential density, and street connectivity [15]. Statistical analysis was conducted to test the impacts of specific measures on obesity. The results indicated that land use mix has the strongest association with obesity. Rutt and Coleman examined the relationship between land use mix and body mass index (BMI), in which a 0.25-mile radius around each person’s residence is defined as neighborhood, to assess transportation and other variables [16]. Eventually, a positive relationship was found between land use mix and BMI. Gordon-Larsen et al. investigated the availability of physical activity and recreational facilities in an 8.05 km (approximate 5 m) buffer area around each participant’s residence [17]. The results indicated that increasing the availability of physical activity and recreational facilities is conducive to lower people’s body weight at the population level. Some studies also employed administrative areas, like census units and postcode areas, to delineate neighborhood [4,7,18,19,20]. For example, Wen and Kowaleski-Jones explored the relationship between the built environment and obesity risk based on census tracts in the United States. The results indicate that attributes of the built environment significantly correlate with obesity risk [20]. Xu et al. examined the associations between built environment factors and individual odds of overweight and obesity at both zip code and county levels [7]. Cobb et al. examined the relationship between local food environments and obesity based on administrative units. The results indicate that density measures based on administrative units are more likely to find expected associations than buffers of less than one mile around individual addresses [21]. Sun et al. examined the influences of the built environment on individual BMI at the county level. It is found that population density and accessibility of facilities are positively related to individual BMI [22]. 

In summary, a common practice in previous studies has been to use static areas, such as census tracts, postcode areas, or buffer areas around people’s residence as contextual units to study the relationship between the built environment and obesity. However, as several researchers in this area have argued in recent years [23,24,25,26], it is inappropriate to use the residential neighborhood or buffers around people’s home location to represent the actual area that exerts contextual influences on people’s health, since individuals move around in their daily life, and are thus exposed to other neighborhoods outside of their residential neighborhood. Thus, how to identify and delineate geographic units that capture individuals’ daily activities and represent their true context is a fundamental challenge in health research on obesity. This challenge has been recently articulated as the uncertain geographic context problem (UGCoP), which is the problem that analytical results can be different for different delineations of contextual units, even if other factors are the same, due to the spatial and temporal uncertainty of the true geographic context [27,28]. Therefore, given that residential neighborhoods may mischaracterize built environment variables for different individuals, this paper seeks to advance our understanding of how different contextual units used to derive environmental variables may affect results in obesity research. It examines the uncertain geographic context problem through analyzing the relationship between the built environment and obesity. Individuals’ residences, workplaces, and primary physical activity locations are simultaneously taken into account while constructing their activity spaces. The focus of this work is to delineate context units using different activity space measures and to examine the relationship between the corresponding built environment variables and obesity for individuals.

## 2. Contextual Uncertainties in Health Research

### 2.1. Neighborhood Effect on Obesity and Physical Activity 

In the past 20 years or so, relations between health and place have been observed for a variety of health behaviors and outcomes at various spatial scales [24,29,30,31,32,33,34]. For instance, Cummins et al. identified several neighborhood features associated with fair to very bad self-rated health (independent of individual factors, such as gender, age, and social class), including poor quality of the physical residential environment, high unemployment, and lower access to private transport [35]. Arcury et al. showed the importance of geographic and spatial behavior factors in rural health care utilization [36]. Based on 40 studies that investigated the associations between neighborhood social environments and coronary heart disease (CHD), Chaix concluded that individuals in at least one population subgroup (e.g., gender, ethnicity) in neighborhoods with deprived socioeconomic positions have an increased coronary heart disease risk, even after controlling for individual-level factors [37]. To conclude, many neighborhood factors exert important contextual influence that directly or indirectly shape health behaviors and outcomes. These factors include physical features of the environment (e.g., walkability, green spaces), the availability and quality of food and other amenities (e.g., grocery stores, health services), the general attractiveness and perceived quality of the neighborhood (e.g., safety, crime), and the level of social organization and support of the local community [38].

Physical activity or obesity is one of the research focuses in health research. There is also considerable research to date that examines the relationship between neighborhood context and physical activity or obesity [39,40,41,42,43]. Many studies used neighborhood disadvantage as a proxy for exposure to, or availability of healthy food options [38]. For instance, Robert and Reither found that increased census tract level disadvantage is associated with higher body mass index (BMI) for women, after taking into account individual-level characteristics such as age, race, individual socioeconomic status (SES), and physical activity [44]. However, results for men indicate no association between BMI and either individual SES or community disadvantage. Using census blocks and block groups as neighborhoods, Boardman et al. found that neighborhoods characterized by high proportions of black residents have a greater prevalence of obesity than areas in which the majority of the residents are white [40]. The association between neighborhood racial composition and obesity, however, is completely attenuated after including statistical controls for the poverty rate and obesity prevalence of respondents’ neighborhoods. Brown et al., observed no direct association between neighborhood type and BMI. However, household heads of single-family dwellings in neighborhoods where respondents made more utilitarian trips by walking or bicycling have lower BMI [45]. Black and Macinko reviewed 37 studies that examined neighborhood determinants of obesity and concluded that the influence of neighborhood-level factors appears mixed [38]. While these studies consistently found that decreased neighborhood-level economic and social resources are associated with high obesity rates, the associations between neighborhood income inequality and racial composition with obesity are mixed. The mixed results, as the authors argued, may be partly due to the different definitions of the neighborhood. For instance, neighborhoods in these studies have been variously delimited based on census tracts, zip code areas, socially and historically defined geographic areas, administrative units, metropolitan statistical areas, and counties. However, these definitions may not adequately operationalize the true geographic context which people are exposed to and interact with. While these studies used administrative areas as contextual units (largely due to the fact that available data on environmental variables are based on these areas), the authors of these studies emphasized that future studies need to provide better justification on how neighborhoods are defined, in order to improve study comparability and clarify the meaning of different neighborhood boundaries and measures. Specifically, the causally relevant contextual area for a particular individual might deviate significantly from any administrative unit. Various neighborhood definitions may lead to different built environment measures and further influence the final results. Therefore, it is necessary to define contextual areas based on the locations of individuals’ daily activities, to take into account the influence of non-residential neighborhoods on their health behaviors or outcomes. 

Similarly, in a study that also considered where subjects shop for groceries, Inagami et al. found that people have higher BMI if they reside in disadvantaged areas and in areas where the average person frequents grocery stores located in more disadvantaged neighborhoods [46]. The study observed that where people shop for groceries and distance traveled to grocery stores are independently associated with BMI. The authors suggested that exposure to grocery stores mediate and suppress the association of residential neighborhoods with BMI, and this may explain why previous studies did not find consistent associations between residential disadvantage and BMI: because they did not account for the shopping behavior of its residents. In a related study, Inagami et al. found that while residence in disadvantaged neighborhoods was associated with worse self-rated health, individuals with greater exposure to less disadvantaged non-residential neighborhoods in their daily activities have better self-rated health [47]. Both studies indicate that exposure to sociogeographic environments, besides where one lives, may modify the impact of the residential neighborhood on health in important ways. As the studies highlight, a person’s activity space is not limited to the residential neighborhood, and it better represents the individual’s environmental and social exposures that are crucial in influencing health outcomes. An important implication is that we need to look beyond the advantage or disadvantage of people’s residential neighborhood (or home census tract) to take into account the advantage or disadvantage of other neighborhoods that may have an impact, either positive or negative, on their health behaviors or outcomes. In particular, instead of stressing that advantage or disadvantage in people’s non-residential neighborhoods may affect their health, this paper emphasizes that people’s activity places (which include both their residential and non-residential neighborhoods) may also influence their health. 

### 2.2. Delineations of Neighborhood Units

As this discussion reveals, neighborhoods in past studies are delineated based on a variety of zones or administrative units, such as census tracts, census block groups, metropolitan statistical areas, or counties [37,38]. Berke et al., for instance, derived objective measures such as land use, land slope, vehicular traffic, and public transit data using neighborhoods delimited with 1 km or 3 km circular zones (buffers) around each respondent’s home [39]. Frank et al., on the other hand, derived objective measures of urban form such as land use mix and residential density, based on neighborhoods delineated with a 1 km road network-based buffer around each participant’s place of residence [48]. For Brownson et al., a neighborhood is defined as a half-mile radius or a 10 min walk from the respondent’s home for some variables, and as a 10-mile radius or a 20 min drive from the respondent’s home for several other variables [49]. 

A possible reason for the mixed results of past studies may thus be the different definitions of neighborhood they used [38]. In addition, these definitions may not adequately operationalize the space where people live and undertake their daily activities and travel. A presupposition of these studies is that the neighborhood of residence or a residence-based buffer area delimited by various means is the most relevant area affecting health behaviors and outcomes, and that neighborhood effects largely operate through interactions among residents of the same neighborhood unit. Another presupposition underlying these delimitations of neighborhood is that individuals who live in the same neighborhood experience the same level of contextual influences, regardless of where they actually live or where they undertake their daily activities and trips. 

Health behaviors and outcomes, however, are related not only to variables derived based on the neighborhood of residence, but also to relevant factors and processes across neighborhoods [29]. The most important determinants of people’s exposure to neighborhood effects or contextual influences are where and how much time they spend while engaged in their daily activities [25,37,50]. People’s activities (and thus exposure to contextual influences) do not take place at one time point and wholly within any static, administrative neighborhood unit. Residential location is only one of the locations where people spend their time, and for most people, the residential neighborhood does not capture the majority of their activities or the locations of these activities [25]. The action space or activity space of individuals is not limited to the residential neighborhood, but may better represent an individual’s environmental and social exposures that affect health behaviors and outcomes [46,47]. Further, exposure to contextual or socioeconomic influences besides where one lives may modify the impact of the residential neighborhood on health in important ways, rendering results among different studies inconsistent.

In light of this, important characteristics of people’s use of and movement across space and time (known as activity space) should also be taken into account when examining the contextual determinants of health behaviors and outcomes [26,38]. These include (1) how much time people actually spend in their residential communities; (2) where else they go, how much time they spend there, and what activities they are involved in when they travel outside of their neighborhoods; (3) what types of areas other residents or peers travel to, and how prevalent and time-extensive these extra-community activities are; and (4) what types of non-residents regularly spend time within the borders of a given local area, and what activities they are engaged in while there [50]. 

This paper seeks to expand conventional notions of neighborhood effects in health research to a broader understanding of sociogeographic context that takes into account where people actually undertake their daily activities. This new concept of context is, in turn, based on the notion of activity space, which is the area containing all locations that an individual has direct contact as a result of his or her daily activities [51]. Because where and when people spend their time differs from individual to individual, the paper argues that this new notion of sociogeographic context will allow us to more accurately evaluate the role of various contextual influences on health behaviors and outcomes for each individual. Since an individual’s activity space can be defined or delineated using different methods, it is important to examine how sociogeographic context, defined and operationalized differently, may influence the analytical results. 

Specifically, this study compares the effects of contextual variables derived from seven different delineations of neighborhood: (1) an individual’s home buffer; (2) an individual’s workplace buffer; (3) an individual’s fitness place buffer; (4) neighborhoods delimited using the standard deviational ellipse; and (5) neighborhoods delimited with the weighted standard deviational ellipse; (6) neighborhoods delimited with the minimum convex polygon; and (7) neighborhoods delimited with road network buffers. In this study, buffers based on an individual’s home, workplace, and fitness place refer to areas around the person’s home, workplace, and fitness place, generated using a specific buffer distance (note that all buffer radii are set to 1 km). Using these seven delineations of neighborhood units, the study moves beyond the neighborhood of residence, and considers the activities and trips that individuals undertake in their daily lives when evaluating people’s exposure to contextual influences. To explore how different definitions of the neighborhood may affect neighborhood effects on health outcomes, the study evaluates the effect of socioeconomic advantage (or disadvantage), derived based on these seven delineations of the neighborhood, on people’s body mass index (BMI).

## 3. Study Area and Data

**Figure 1:**
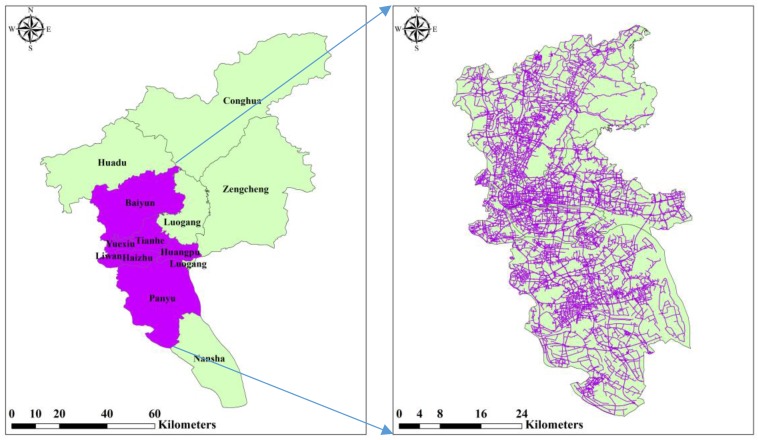
The administrative units of Guangzhou city and study area.

The study area of this research is located within the city of Guangzhou, which is the capital of Guangdong Province in China and one of the country’s major cities. The city had a population of approximately 12.7 million in 2010. Its total area is 7434.4 square kilometers. In this study, the peripheral zone outside the beltway in Guangzhou (as shown in Figure 1) is selected as the study area, which includes the central, transition, and marginal districts [52,53]. This area is chosen based on its history, location, and housing types (e.g., traditional self-built housing, welfare housing, *danwei* compounds, mixed residential areas, commercial housing, and urban villages). It includes seven urban districts: Liwan, Panyu, Tianhe, Haizhu, Huangpu, Baiyun, and Yuexiu. 

As the main urban areas in Guangzhou, these seven districts have different population density and resident income. For example, census data show that the population density of Yuexiu district is 33,920 per square kilometer, while the population density of Huangpu district is 1856 per square kilometer in 2015 the same year. Areas of higher population density tend to have a higher proportion of the population that use active transportation, as destinations in these areas are closer together and can often be reached by walking or bicycling [54]. More importantly, these highly urbanized districts often account for higher proportions of public facilities (e.g., schools, hospitals, and shopping malls) when compared to other areas such as suburbs. However, differences also exist among these districts. For instance, the main urban zones have approximately 80% of the large and medium-sized medical institutions in the city, of which 50% of the ministerial, provincial, and municipal medical institutions are located in Yuexiu district. These urban characteristics of the study area may affect the participants’ physical activity and body weight. Geographic data of the study area, including road networks data and points of interest (POI) data in 2015, are used in the study to calculate the built environment variables. 

## 4. Methods

This study seeks to examine how various built environment variables derived with different delineations of contextual areas affect their influence on people’s body weight. It uses body weight (implemented with the body mass index) as the health outcome or dependent variable. It uses five built environment variables as the contextual influences or independent variables: residential density, land use mix, street density, fast food restaurants density, and transit stations density. Various delineations of contextual areas are implemented. These are the standard deviational ellipse (SDE), the weighted standard deviational ellipse (WSDE), the minimum convex polygon (MCP), the road network buffer (RNB), the workplace buffer (WPB), and the fitness place buffer (FPB), which are compared with the residential buffer (note that all buffer radii are set to 1 km). Through delineating individuals’ contextual areas using various activity spaces, we examine how built environment variables derived with different activity spaces may influence the association between physical activity and body weight. Participants’ weight status in this study is assessed by the body mass index (BMI), which is calculated based on height and weight of actual measurement provided in the survey data: BMI = weight(kg)/(height (m))^2^. According to Flegal et al. [55], an adult of 20 years old or older, who has a BMI between 25.0 and 29.9, is considered overweight, and a BMI of 30 or higher is defined as obese.

### 4.1. Social Survey

The original individual-level data used in the study were collected from survey questionnaires based on a random sample of households in Guangzhou in January 2016. The survey sought to examine residential and employment change, as well as health and medical care of the city’s residents. Information collected from the participants includes personal information (including weight and height), residential and employment change, personal fitness, lifestyle habits, health care, and community environment. Thirty-six communities in 11 neighborhoods with the size of approximately 1 km^2^ were selected from all of the administrative districts in Guangzhou. The detailed selection process is described in the literature [53]. A total of 1029 returned questionnaires were valid and usable. 

Note that the numbers of respondents’ activity locations recorded in the survey are different. There are 1029 homes, 1011 workplaces, and 784 fitness places. In addition, the fitness places where moderate-to-vigorous physical activities were performed for several respondents are the same as their home location or their other recorded fitness locations. In this study, since constructing several activity space measures (e.g., standard deviational ellipse and minimum convex polygon) require three or more activity places, we only include the respondents whose residential, workplace, and fitness places are not the same place, to ensure that the possibility of deriving all activity space measures. Eventually, 403 respondents meet this condition and were included in the analysis. 

Table 1 summarizes the socioeconomic characteristics of the participants, whose gender, age, education, income, and marital status are described. The proportions are closely balanced between men and women. More than 75% of the participants are under the age of 50, in which juveniles under 18 are excluded. The majority of the residents’ level of education is high school or below. Income is divided into five levels, in which participants with income less than 15,000 Yuan account for 60%. Marriage is summarized as single, married, and divorced status, in which married status occupies the major proportion.

### 4.2. Built Environment Variables

As mentioned above, insufficient physical activity and excessive energy intake contributes to individual’s excessive body weight and may lead to obesity. In this study, built environment variables are delineated based on these two aspects, namely, neighborhood walkability, and fast food outlets. Numerous studies have indicated that better walkability tends to associate with higher levels of physical activity, and easy access to fast food outlets usually enhance extra calorie intake. For instance, if individuals’ residences are close to activity destinations, such as public transit stations or to areas with higher street density, they tend to undertake more walking or bicycling. Neighborhood walkability is often conceptualized via the three Ds: density, design, and diversity [4,56]. Density provides a large collection of people, which can be measured by population or residential density. Design and diversity are often measured by street density and mixed land use, respectively. Transit station density can be used to measure accessibility. In this paper, five built environment variables closely associated with individual’s body weight are selected as the contextual variables: residential density, land use mix, street density, fast food restaurant density, and transit station density. 

Residential density (RD) is commonly defined as the density of residences within an individual’s 1 km home buffer [34,48]. Areas with greater residential density are normally considered more walkable than areas with lower residential density. In addition, if an area has a higher residential density, it is often more mixed and interconnected and more likely to promote physical activity [48]. In this study, RD is defined as the density of residences within a participant’s activity space.

Land use mix (LUM) can be calculated using a variation of entropy index, where the proportion of land use types, including commercial, residential, office, and entertainment in an area, are taken into account [41,48,57]. In this study, the area is a participant’s activity space. The values of land use mix range from 0 to 1, which measures how evenly the proportion of commercial, residential, office, and entertainment area is distributed within each area. The values 0 and 1 correspond to a single land use environment and one with the greatest land use heterogeneity, respectively. Previous studies have shown that physical activity can also be facilitated by higher land use mix, since it provides a variety of destinations within walking distance, such as restaurants, parks, and transit stations. Therefore, land use mix tends to encourage more walking. The formula for land use mix is shown as follows:

(1) $$H=-1\left(\sum_{i=1}^{n}p_{i}*\ln\left(p_{i}\right)\right)/\ln\left(n\right)$$

where H is the land use mix score, $p_{i}$ is the proportion of land use i among all land use classes, and n is the number of land use types.

Street density (SD) is calculated as road or street length (in kilometer) divided by land area within 500 m and 1000 m buffers, which can be used to measure neighborhood design characteristics and reflect road network connectivity [58]. It has also been shown that street density is closely related to physical activity. Higher density of street networks and more densely-connected street networks represent shorter distances between destinations, which is conducive to walking trips [59]. In this study, we define street density (SD) as the total length of the street or road segments within a participant’s activity space divided by the area of the activity space.

Fast food restaurant density (FFRD) is the number of fast food restaurants within a participant’s activity space divided by the area of the activity space. Here, fast food restaurants are defined as food outlets that prepare and serve mass-produced food very quickly, and they include both Western and Chinese style fast food outlets. A growing number of studies indicated that food consumption tied to fast food restaurants is more likely to enhance higher caloric intake. FFRD reflects the accessibility of high-energy diets to a certain extent. If fast food restaurants are located near an individual’s home, he/she tends to consume more high-energy meals. It has also been found that better access to fast food is closely associated with a higher likelihood of obesity [60].

Transit station density (TSD) refers to the number of transit stations per square kilometer in an area. In this study, this area is the area of a participant’s activity space. As the primary mode of access to transit, walking is also closely related to transit station density, and more use of transit often corresponds to more walking when compared to driving. Past research indicates that participants in areas with high transit station density are more likely to use transit than those located within the same distance from transit stations in areas with low transit station density [61]. 

### 4.3. Delineating Individuals’ Contextual Areas Using Activity Space

As discussed above, traditional contextual units, such as census tracts or postcode areas, may not accurately represent individuals’ activity spaces, since they ignore individual’s mobility, spatial habits, and travel environment [27,28,62]. In this study, activity space is used to delineate individuals’ contextual units. It is the area within which an individual undertakes or travels to his/her actual daily activities [62,63]. Activity space can be expressed as a two-dimensional area that covers the spatial distribution of the locations an individual visited. Several factors determine the geometry, size, and structure of an individual’s activity space. These include the home location, the location of the workplace, the locations of regular activities, and the daily movements between these locations and activities. Therefore, activity spaces are often delineated by many activity locations of an individual, or based on individual’s actual travel trajectory when detailed movement data are available. 

In this paper, the survey data mainly contain participants’ three activity destinations: home, workplace, and a fitness place (the primary location where the participant undertakes physical activity). Note that while each participant may perform several physical activities, only the location for their primary physical activity (called the fitness place in this study) is included in this analysis. The activity space measures for this study are thus constructed based on these three places. Although the activity spaces delineated in this study may not fully represent the participants’ actual activity spaces due to this data limitation, they are still more accurate when compared to conventional delineations of contextual units based only on the home or working place location, since they not only consider both of these locations but also take the primary location of physical activity into account. 

Considering that different activity spaces may yield different results, this paper presents four different activity space delineations, including the standard deviational ellipse, the weighted standard deviational ellipse, the minimum convex polygon, and road network buffer. They are used to study the relationship between environmental variables and participants’ BMI, and they are also compared with the results based on participant’s home buffers, workplace buffers, and fitness place buffers.

#### 4.3.1. The Standard Deviational Ellipse

**Figure 2:**
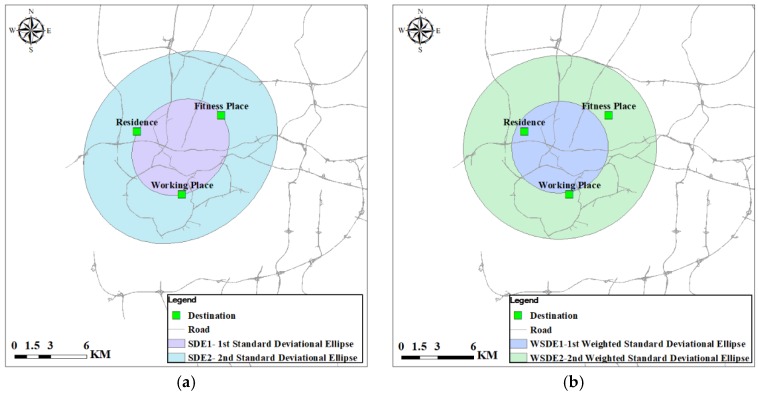
Examples of the standard deviational ellipse and weighted standard deviational ellipse. (**a**) Standard deviational ellipse; and (**b**) weighted standard deviational ellipse.

The standard deviational ellipse (SDE) measures the spatial distribution of activity destinations and can be calculated using the CRIMESTAT spatial statistics package [64]. The long axis and short axis of the SDE correspond to the direction of maximum dispersion and minimum dispersion, respectively, among the points. The SDE has been used to measure individuals’ travel behaviors in urban environments [63,65]. It has been implemented as one standard deviational ellipse (SDE1) and/or two standard deviational ellipse (SDE2), which cover approximately 68% and 95% of all activity points, respectively (if all points have equal weights). As shown in Figure 2a, activity space of a single respondent is represented with standard deviational ellipses, including SDE1 and SDE2. 

Generally speaking, the numbers of times per year an individual visits different destination are different. We further construct the weighted standard deviational ellipse based on visit frequencies in this study. Activity destinations are weighted based on the number of times per year the destinations are visited by the individual. Since the survey data only include the visit frequency of fitness places, the visited frequencies of home and workplace are set based on the following assumptions. The weighted value is set to 365 for the residence, assuming that individuals go home every day. Working places are weighted by the value 260 (5 times for 52 weeks), assuming that individuals work five times per week. For fitness place, weighted values are determined based on the data provided by participants in the survey. Here, we use the respondent in Figure 2a as an example to explicate the construction of weighted standard deviational ellipse (WSDE). The visit frequencies to the residence, workplace and fitness place are 365, 260, 208 respectively. The weighted standard deviational ellipse can be obtained on the basis of these frequencies, as displayed in Figure 2b. Considering that SDE1 just covers 68% of activity points and only three activity points are used to construct the standard deviational ellipse, several participants’ activity space may not be constructed using SDE1. Therefore, this study selects SDE2 and WSDE2 to delineate activity space.

#### 4.3.2. The Minimum Convex Polygon

**Figure 3:**
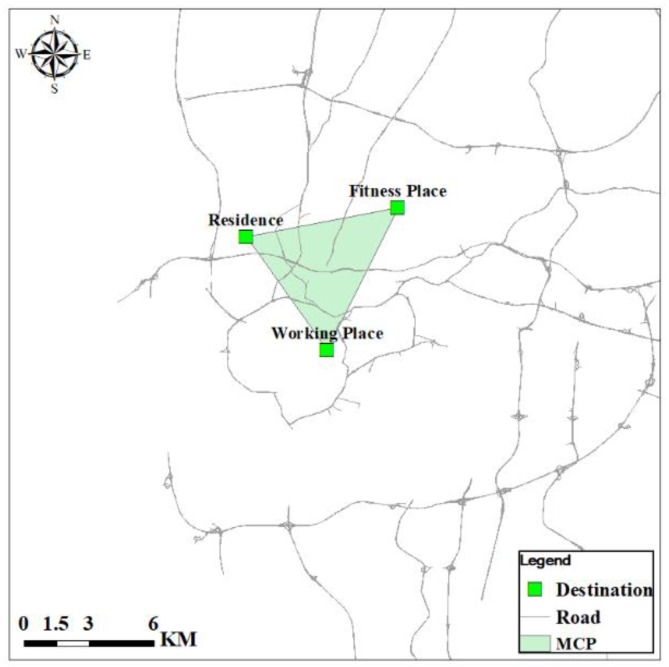
Example of the minimum convex polygon.

The minimum convex polygon (MCP) is the smallest convex polygon that covers a set of points [63]. The MCP is straightforward to construct using ArcGIS 10.1 ArcToolbox, which can be used to identify the contexts related to individual’s BMI. Since only three destinations are included in the survey, the MCPs constructed in this study are triangles. As shown in Figure 3, the activity space of a single respondent is represented by the MCP, which covers the respondent’s residence, workplace, and fitness place.

#### 4.3.3. The Road Network Buffer

**Figure 4:**
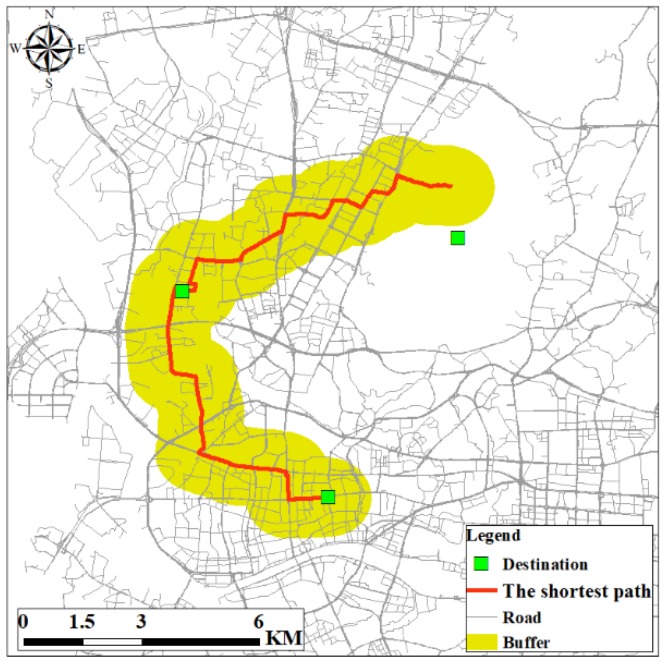
Example of road network buffer.

The road network buffer (RNB) is constructed based on the roads used by an individual to travel among the home, workplace, and other activity locations [62]. First, the shortest paths between respondents’ residence and the other two destinations are calculated using the ArcGIS Network Analyst Extension. Then, a buffer is obtained around the shortest paths. However, two limitations exist in the construction of the RNB. The first limitation is that the quality of the RNB is closely related to the road network data. The second limitation is that the shortest paths between home and other destinations are not necessarily the respondent’s actual routes. The selection of buffer size is also crucial for constructing the RNB. Previous studies indicated that 1 km buffers are appropriate [48,62]. In this paper, we also choose 1 km as the buffer size for constructing the RNB. Figure 4 displays the RNB for the same respondent in Figure 2 and Figure 3. It indicates that the shape, area, and extent of RNB are different from SDE and MCP.

### 4.4. Statistical Analysis

The statistical analysis of different activity space measures has two main objectives. The first is to compare the contextual variables obtained with different activity space delineations in order to examine whether they are significantly different. A 1-km buffer area is created around each participant’s home location. Paired sample *t*-tests are utilized to examine whether significant differences exist between activity space based contextual variables, and those obtained using the home buffers. Second, regression analysis is conducted using multivariate linear regression, which takes the built environment variables and individual variables as the independent variables, and BMI as the dependent variable. The purpose is to analyze the effect of environmental and sociodemographic variables on participants’ BMI. Previous related studies also demonstrate that age has close associations with obesity [66]. The model is as follows:

(2) $$\begin{array}{cc} BMI= & \beta_{0}+\beta_{1}RD+\beta_{2}LUM+\beta_{3}SD+\beta_{4}FFRD+\beta_{5}TSD\\ & +\beta_{6}G+\beta_{7}A+\beta_{8}E+\beta_{9}I+\beta_{10}M+\varepsilon \end{array}$$

The independent variables of the model are explained in Table 2.

## 5. Analysis

### 5.1. Comparative Analysis of Activity Space Measures

We compare the activity space measures with the home buffer, working place buffer, and fitness place buffer, respectively, with respect to two aspects: attributes of various delineations of activity space, and values of the contextual variables derived with them. First, areas of the activity space are compared in order to understand the differences between the measures (see Table 3). Since the buffer sizes of each respondent’s home buffer are identical, the home buffers, workplace buffers, and fitness place buffers have the same mean, median, and maximum area (3.14 km^2^). Among the various contextual areas, the home buffers, working place buffers, and fitness place buffers have the smallest mean area (3.14 km^2^) and the smallest maximum area (3.14 km^2^), while SDE2 has the largest mean area (43.96 km^2^) and the largest maximum area (1135.75 km^2^). The MCP has the smallest median area (1.38 km^2^), while RNB has the largest median area (23.68 km^2^). Compared with SDE2, WSDE2 has lower mean, median, and maximum area, respectively.

**Figure 5:**
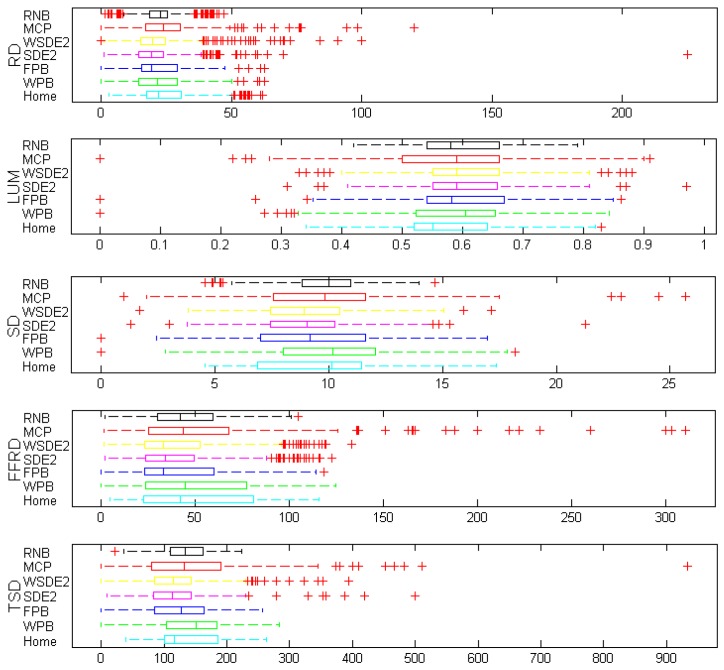
Distribution of normalized built environment measures. Boxes represent the interquartile range, whiskers represent the range of values, and crosses represent the outliers.

Further, we analyze the range, median, and interquartile range of the built environment variables based on different activity measures. Figure 5 shows the distribution of the environmental variables derived with different activity space delineations using boxplots. It indicates that the built environment measures have various distribution characteristics due to different shapes and sizes of the contextual areas. For instance, the values of RD derived with road network buffers have a range of 0–50, while those of WSDE2 have a range between 0 and 100.

Next, we performed paired sample *t*-tests to examine whether significant differences are evident in the built environment variables obtained from different activity space delineations, and from the home buffers. Table 4 summarizes the significance of paired sample *t*-test for built environment variables between the activity space measures for the 403 participants. A significance level of 0.05 is used to judge whether two measures are significantly different. As shown in Table 4, significant differences exist for the built environment variables for most of the pairs of contextual areas. However, there are also several pairs of contextual areas with no significant differences for the built environment variables. For instance, there are no significant differences for residential density (RD) between measures derived with the following ten pairs of contextual areas: WPB and FPB, WPB and SDE2, WPB and WSDE2, WPB and RNB, FPB and SDE2, FPB and WSDE2, FPB and RNB, WSDE2 and SDE2, WSDE2 and RNB, and MCP and the home buffers. For land use mix (LUM), significant differences are non-existent between measures derived with WPB and home buffers, FPB and MCP, WSDE2 and SDE2. Significant differences are non-existent for street density (SD) between measures derived with the following five pairs of contextual areas: FPB and SDE2, FPB and WSDE2, WSDE2 and SDE2, MCP and RNB, and MCP and the home buffers. For fast food restaurant density (FFRD), significant differences exist for measures derived with the following five pairs of contextual areas: WPB and home buffers, FPB and RNB, WPB and MCP, WSDE2 and SDE2, and MCP and the home buffers. Lastly, significant differences exist for transit station density (TSD) between measures derived with the following six pairs of contextual areas: WPB and home buffers, WPB and MCP, FPB and SDE2, WSDE2 and SDE2, MCP and the home buffers, and RNB and the home buffers. In addition, all pairs of the built environment variables derived with WSDE2 and SDE2 have no significant difference. Four of the five built environment variables derived with MCP and the home buffers are not significantly different. These differences may affect research findings concerning the influence of environmental variables on people’s BMI. We explore this possibility in what follows. 

### 5.2. Regression Analysis of Obesity

In this section, regression analysis is conducted to explore how built environment variables derived with different contextual areas influence participants’ BMI. In order to further demonstrate whether individuals’ activity space influences the results, we also compare the regression analysis results based on the home buffers, WPB, and FPB with that of other activity space measures. 

Table 5 summarizes the results of the regression analysis based on different activity space measures, including home buffers, WPB, FPB, SDE2, WSDE2, MCP, and RNB. Coefficients with an asterisk mean that the corresponding independent variables are significant in the regression models. The regression results indicate that the selection of activity space measures may influence which built environment variables have significant effects on obesity. On the one hand, different variables may be significant in models using variables derived with different contextual areas. For instance, significant built environment variables are street density, fast food restaurant density, and transit station density in the model using environmental variables derived with home buffers, while significant built environment variables for models that used variables derived with SDE2 and RNB are land use mix and transit station density, respectively. Built environment variables are not significant in the models that used variables based on WSDE2 and MCP. The regression coefficients reflect the relationships between the variables and participants’ BMI. For the model that used environmental variables derived with home buffers, the coefficients of SD, FFRD, TSD (−0.183, 0.305, and −0.330) indicate that street density and transit station density are negatively associated with BMI, while fast food restaurant density is positively related with BMI. 

In contrast, the demographic variables of age, income, and marital status are significant for all the models. Further, their relationships with participants’ BMI are consistent for all of the seven regression models. Higher income is associated with lower BMI, and being older and being married are associated with higher BMI. On the other hand, values of the adjusted R^2^ are different for the regression models, as shown in Table 4. The different R^2^ values represent the different explanatory power of these models. For instance, the adjusted R^2^ (0.202) of the model that used variables derived with home buffers indicates the variables explain 20.2% of the variance of the dependent variable (BMI). We also calculate the variance inflation factor (VIF) values of each variable for all models, which are all far less than 10. This suggests that there is no multi-collinearity among the independent variables. In addition, we assess the significance of each regression model using the F test. The results indicate that all the models are significant at the 0.05 significant level. In other words, there is a significant linear relationship between the dependent variable and the independent variables.

## 6. Discussion

Overall, the research findings highlight the existence of the uncertain geographic context problem when examining the relationships between the built environment and obesity. In other words, whether an environmental variable has a significant influence on participants’ BMI depends on the contextual areas used to derive it. The aim of this study is not to determine which activity space delineation should be used, but to apply the concept of activity space to illustrate the uncertain geographic context problem through analyzing the relationships between a set of built environment variables and obesity. After all, people are not fixed to a single location in their daily life, and it is inadequate to delineate their activity space with any fixed location [62]. The results indicate that the selection of activity space measures influence whether an environmental variable affects obesity. A few previous studies also demonstrated the effect of geographic context on health. For example, Boone-Heinonen et al. analyzed the impact of buffer sizes on moderate and vigorous physical activity (MVPA) and found that the measures within buffers with different radii had different relationships with MVPA [67]. James et al. studied the effects of buffer size and shape on associations between the built environment and people’s energy balance, and demonstrated that the scale and shape of buffers influenced the results [59]. The results of these studies indicate the importance of the uncertain geographic context problem in health research and illustrate this problem by delineating geographic context using various activity spaces.

Our analysis examines the associations between built environment variables and obesity for different activity space measures. Although the coefficients of the built environment variables based on participants’ home buffers are slightly higher than those based on other neighborhood or activity space delineations, it does not indicate that built environment variables based on home buffers are the best measures for analyzing the impact of the built environment on obesity. Specifically, the home buffers model is not suitable for delineating all the environmental variables. It is worth noting that since the environmental variables of street density (SD) and fast food restaurant density (FFRD) have a significant effect on participants’ BMI for the home buffers model, but do not have a significant effect on participants’ BMI for other activity space delineations. For instance, FPB and SDE2 are suitable to analyze the influence of residential density (RD) and land use mix (LUM) on obesity, respectively. The results support conclusions made by other related studies that perhaps there is no one “best” single measure for depicting people’s activity space based on a small number of activity places [59,62,65,68]. For the higher coefficient of the home buffer model, it is possible that the residential neighborhood may be the most influential environment in which participants’ daily activities are conducted, and much of their environmental exposures are experienced. For instance, high density of road network and bus stops around people’s residences are more likely to encourage residents to give up traveling by motor vehicles. Besides, large numbers of fast food restaurants around people’s residences may attract them to select fast food. 

In addition, we also compare the effects of contextual variables derived from seven activity space measures, including home buffers, WPB, FPB, SDE2, WSDE2, MCP, and RNB. However, four of these measures, namely SDE2, WSDE2, MCP, and RNB, combine home, workplace, and fitness place into one large activity space, which may not be able to differentiate the activities that people undertake in these areas. The activity spaces based on each of these areas separately may facilitate us to compare the impact of the different built environments of different areas on people’s health. The results from Table 4 and Table 5 demonstrate that there are some significant differences in the relationships between built environment variables and obesity based on activity spaces that centered on one area, and on activity spaces that combined three areas (residence, workplace, and fitness place).

## 7. Conclusions

The objective of this study is to examine the uncertain geographic context problem when analyzing the associations between obesity and the built environment. The uncertain geographic context problem emphasizes that the impact of the precise geographic delineations of contextual units or the deviation of the contextual units from the actual geographic context on analytical results. However, previous studies mainly focused on analyzing the associations between obesity and built environment variables based on static geographic units [5,7,20]. Little attention has been paid to accurately capturing individuals’ exposure to environmental influences when studying obesity. As far as we know, this study is one of the first to examine associations between obesity and built environment variables via delineating individuals’ actual activity space.

Using survey and GIS data of Guangzhou, five built environment variables are computed: residential density, land use mix, street density, fast food restaurant density, and transit station density. These variables are derived using seven different contextual areas, including home buffers, WPB, CPB, SDE2, WSDE2, MCP, and RNB. In addition, sociodemographic variables such as gender, age, education, income, and marital status are included. Different activity space delineations are compared, and the results show that differences between activity space sizes are evident. In addition, we compare the built environment variables obtained with different activity space measures using paired sample *t*-tests. The test results indicate that significant differences exist between several activity space based built environment variables. On the other hand, we analyze associations between obesity and the built environment and individuals’ sociodemographic variables based on different activity space measures using multivariate linear regression models. It is found that individuals’ activity space delineations have a significant influence on analytical results. The relationships between obesity and the built environment are influenced by the contextual areas used to derive the environmental variables. Finally, it is noteworthy that age, income, and marital status play an important role in obesity in all models. 

In conclusion, the UGCoP as a fundamental problem in health research calls for continuous concerns on its confounding effects and mitigation. The present study sheds light on the UGCoP in analyzing the relationships between obesity and the built environment. Nevertheless, two limitations of this research need to be acknowledged. First, only the home, workplace, and primary physical activity location for each participant are used to delineate individual activity space in this study due to data limitation. Using more complete activity–travel data of subjects (e.g., collected with GPS) may be helpful in more accurately representing participants’ activity space. Second, previous studies indicate that demographic variables are important moderators of the relationships between obesity and environmental variables. This study only considers a small number of respondents’ sociodemographic characteristics as control variables. Future research is warranted to investigate how other demographic variables may influence analytical results.

## Figures and Tables

**Table 1:** Individual’s socioeconomic characteristics.

Socioeconomic Variables		Number of Participants	Percentage
Gender (G)	Men	203	50.4%
	Women	200	49.6%
Age (A)	18–29	91	22.6%
	30–40	110	27.3%
	41–50	114	28.3%
	51–65	46	11.4%
	65+	42	10.4%
Education (E)	High school or below	165	40.9%
	Technical school	59	14.6%
	Junior college	103	25.6%
	College or high	76	18.9%
Income (I)	Less than ¥ 15,000	245	60.8%
	¥ 15,000 to less than ¥ 20,000	98	24.3%
	¥ 20,000 to less than ¥ 30,000	46	11.4%
	¥ 30,000 to less than ¥ 50,000	9	2.2%
	¥ 50,000 or more	5	1.3%
Marriage (M)	Single	78	19.4%
	Married	323	80.1%
	Divorced	2	0.5%

**Table 2:** Explanations of the abbreviations for the independent variables.

Abbreviations	Independent Variables
RD	Residential density
LUM	Land use mix
SD	Street density
FFRD	Fast food restaurant density
TSD	Transit station density
G	Gender
A	Age
E	Education
I	Income
M	Marriage

**Table 3:** Area of activity space measures.

Activity Space	Mean Area (km^2^)	Median Area (km^2^)	Maximum Area (km^2^)
Home Buffers	3.14	3.14	3.14
WPB	3.14	3.14	3.14
FPB	3.14	3.14	3.14
SDE2	43.96	13.18	1135.75
WSDE2	30.98	9.71	576.77
MCP	4.59	1.38	117.34
RNB	26.06	23.68	108.48

(Home Buffers = buffers around home; WPB = buffers around workplace; FPB = buffers around the fitness place; SDE2 = standard deviational ellipse at two standard deviations; WSDE2 = weighted standard deviational ellipse at two standard deviations; MCP = minimum convex polygon; RNB = road network buffer).

**Table 4:** Significance of paired sample *t*-test for built environment variables between the activity space measures.

		Home	WPB	FPB	SDE2	WSDE2	MCP	RNB
**RD**	Home		0.003 *	0.000 *	0.000 *	0.000 *	0.563	0.000 *
	WPB	0.003 *		0.767	0.208	0.869	0.000 *	0.302
	FPB	0.000 *	0.767		0.316	0.863	0.000 *	0.084
	SDE2	0.000 *	0.208	0.316		0.185	0.000 *	0.010 *
	WSDE2	0.000 *	0.869	0.863	0.185		0.000 *	0.146
	MCP	0.563	0.000 *	0.000 *	0.000 *	0.000 *		0.001 *
	RNB	0.000 *	0.302	0.084	0.010 *	0.146	0.001 *	
**LUM**	Home		0.746	0.005 *	0.000 *	0.000 *	0.000 *	0.001 *
	WPB	0.746		0.041 *	0.001 *	0.002 *	0.008 *	0.025 *
	FPB	0.005 *	0.041 *		0.000 *	0.000 *	0.503	0.000 *
	SDE2	0.000 *	0.001 *	0.000 *		0.396	0.000 *	0.002 *
	WSDE2	0.000 *	0.002 *	0.000 *	0.396		0.000 *	0.013 *
	MCP	0.000 *	0.008 *	0.503	0.000 *	0.000 *		0.000 *
	RNB	0.001 *	0.025 *	0.000 *	0.002 *	0.013 *	0.000 *	
**SD**	Home		0.003 *	0.000 *	0.000 *	0.000 *	0.447	0.025 *
	WPB	0.003 *		0.000 *	0.000 *	0.000 *	0.031 *	0.011 *
	FPB	0.000 *	0.000 *		0.822	0.794	0.000 *	0.000 *
	SDE2	0.000 *	0.000 *	0.822		0.930	0.000 *	0.000 *
	WSDE2	0.000 *	0.000 *	0.794	0.930		0.000 *	0.000 *
	MCP	0.447	0.031 *	0.000 *	0.000 *	0.000 *		0.421
	RNB	0.025 *	0.011 *	0.000 *	0.000 *	0.000 *	0.421	
**FFRD**	Home		0.974	0.001 *	0.000 *	0.000 *	0.171	0.000 *
	WPB	0.974		0.004 *	0.000 *	0.000 *	0.211	0.000 *
	FPB	0.001 *	0.004 *		0.001 *	0.009 *	0.000 *	0.442
	SDE2	0.000 *	0.000 *	0.001 *		0.064	0.000 *	0.000 *
	WSDE2	0.000 *	0.000 *	0.009 *	0.064		0.000 *	0.000 *
	MCP	0.171	0.211	0.000 *	0.000 *	0.000 *		0.000 *
	RNB	0.000 *	0.000 *	0.422	0.000 *	0.000 *	0.000 *	
**TSD**	Home		0.262	0.000 *	0.000 *	0.000 *	0.201	0.073
	WPB	0.262		0.000 *	0.000 *	0.000 *	0.733	0.001 *
	FPB	0.000 *	0.000 *		0.094	0.019 *	0.000 *	0.000 *
	SDE2	0.000 *	0.000 *	0.094		0.240	0.000 *	0.000 *
	WSDE2	0.000 *	0.000 *	0.019 *	0.240		0.000 *	0.000 *
	MCP	0.201	0.733	0.000 *	0.000 *	0.000 *		0.025 *
	RNB	0.073	0.001 *	0.000 *	0.000 *	0.000 *	0.025 *	

* Significance level at *p* < 0.05.

**Table 5:** Results of multiple linear regression based on different activity space measures.

Multiple Linear Regression Models							
	**Home Buffer**	**WPB**	**FPB**	**SDE2**	**WSDE2**	**MCP**	**RNB**
	(R^2^ = 0.218)	(R^2^ = 0.178)	(R^2^ = 0.184)	(R^2^ = 0.183)	(R^2^ = 0.182)	(R^2^ = 0.176)	(R^2^ = 0.188)
	**Coefficient**	**Coefficient**	**Coefficient**	**Coefficient**	**Coefficient**	**Coefficient**	**Coefficient**
**NRD**	0.135	−0.026	0.155 *	−0.09	0.007	0.009	0.091
**LUM**	−0.07	−0.062	−0.002	0.123 *	0.088	0.07	−0.066
**SD**	−0.183 *	0.056	0.012	−0.032	−0.058	−0.034	0.002
**FFRD**	0.305 *	0.024	−0.127	0.069	0.053	0.021	0.238
**TSD**	−0.33 *	−0.082	0.005	−0.013	−0.02	0.014	−0.315 *
**G**	−0.06	−0.064	−0.061	−0.056	−0.063	−0.067	−0.079
**A**	0.301 *	0.233 *	0.272 *	0.269 *	0.268 *	0.258 *	0.264 *
**E**	−0.085	−0.112	−0.090	−0.101	−0.106	−0.096	−0.116
**I**	−0.152 *	−0.117 *	−0.136 *	−0.138 *	−0.147 *	−0.127 *	−0.141 *
**M**	0.107 *	0.145 *	0.138 *	0.128 *	0.128 *	0.129 *	0.131 *

* Coefficient significant at *p* < 0.05.
